# *Wolbachia* density changes seasonally amongst populations of the pale grass blue butterfly, *Zizeeria maha* (Lepidoptera: Lycaenidae)

**DOI:** 10.1371/journal.pone.0175373

**Published:** 2017-04-12

**Authors:** Takuto Sumi, Kazuki Miura, Takahisa Miyatake

**Affiliations:** 1 Laboratory of Evolutionary Ecology, Graduate School of Environmental and Life Science, Okayama University, Kita-ku, Okayama, Japan; 2 National Agriculture and Food Research Organization Western Region Agricultural Research Center, Fukuyama, Japan; Centro de Pesquisas René Rachou, BRAZIL

## Abstract

Previous studies showed that the survival rate of *Wolbachia* decreases under high temperature in incubators. It is also known that a high density of *Wolbachia* in the host body reduces the host emergence rate, while low densities fail to change reproduction rates. However, few studies have examined the density of *Wolbachia* in hosts in the field. Here, we focus on *Wolbachia* infection of the pale grass blue butterfly, *Zizeeria maha* (Lepidoptera: Lycaenidae), which is distributed throughout the Japanese islands. We examined the rate and density of *Wolbachia* infection in the bodies of butterflies at thirteen locations in Japan. At seven of these places, we collected butterflies in different seasons to determine seasonal differences in the infection rate and density and found that *Wolbachia* density has seasonal differences within the same population. Moreover, to determine whether *Wolbachia* density has a geographical cline, we compared the infection density of *Wolbachia* amongst all geographical populations. In addition, we determined the sequences of *Wolbachia wsp* and host mtDNA *CO1* haplotypes of all populations. The results showed that *Wolbachia* density increased in early summer and decreased in autumn. Further, the density of *Wolbachia* infecting the same strain of *Z*. *maha* varied amongst populations, although no tendency in geographical cline was observed.

## Introduction

*Wolbachia* are endosymbionts belonging to the alpha-proteobacteria [1]. This bacterium is transmitted from mother to progeny [1] and causes a variety of reproductive manipulations in its hosts [2]. These manipulations were classified into four types; feminization of males, induction of thelytokous parthenogenesis, male killing, and cytoplasmic incompatibility (CI) [1–3]. Of these phenomena, CI is the most common manipulation by *Wolbachia*. CI affects sperm [4], and thus the infected males play an important role in the infected population. The eggs derived from an infected male and an uninfected female do not hatch. As a result, *Wolbachia*-infected individuals dominate the host population.

External environmental conditions significantly affect the infective states of hosts including the density of the endosymbionts inhabiting the host body. For example, high temperatures are not salubrious for *Wolbachia*, even temperatures that they are normally exposed to in the fields. Some studies showed that high temperatures eliminated *Wolbachia* from *Drosophila melanogaster*, *Tetranychus urticae*, and other species, but these studies were conducted in the laboratory [3,5–8]. Moreover, the density of *Wolbachia* infecting host bodies affects the success of the reproductive manipulation. In *Culex pipiens*, high *Wolbachia* density decreases the host emergence rate [9], but low *Wolbachia* density fails to manipulate host reproduction in *Drosophila innubila* [10].

Laboratory studies have shown that different geographical strains of some insects have different densities of *Wolbachia*. For examples, Mouton et al. (2007) compared *Wolbachia* densities between progenies of crosses of strains of a wasp with different densities of *Wolbachia* [11]. The F1 hybrid showed the mean *Wolbachia* density of their mothers. This suggests that *Wolbachia* density is maintained by the host genotype. On the other hand, it has been suggested that *Wolbachia* density changes with the season [10]. In addition, Unckless et al. (2009) suggested that *Wolbachia* density changes with the age of the host [10]. However, little is known about whether these phenomena occur in nature. Although Toju and Fukatsu (2011) reported that the *Wolbachia* infection rate in *Curculio sikkimensis* has a geographical cline, they did not reveal whether *Wolbachia* density has a geographical cline [12].

Previous laboratory studies suggested that infection rates and density of *Wolbachia* differ in host populations that inhabit temperate zones in summer [13]. In the present study, we hypothesized the existence of a geographical cline of *Wolbachia* density. This hypothesis expects that the abundance of *Wolbachia* is terminated or decreases in the host body in hot temperature regions, and *Wolbachia* density thereby varies amongst geographical populations. To verify this hypothesis, *Zizeeria maha* (Lepidoptera; Lycaenidae) is an ideal subject because the *Wolbachia* infection status has been studied in this butterfly, which inhabits the main island of Japan from north to south [13]. This makes it possible to verify the *Wolbachia* infection status of the butterfly in geographically separated populations. Furthermore, it is easy to compare the *Wolbachia* density of wild-caught butterflies, which are obtainable from spring to autumn. In the present study, we collected *Z*. *maha* butterflies across different latitudes and longitudes, and verified the rates of *Wolbachia* infection and density for the different populations using qPCR.

Studies of *Wolbachia* using multilocus sequence typing (MLST) have demonstrated the power to discriminate between *Wolbachia* strains [14]. Here, we also conducted MLST analysis and reproductive manipulation experiments to identify and characterize the effects of the *Wolbachia* strain that infects *Z*. *maha*.

## Material and methods

### Sampling

**Fig 1:**
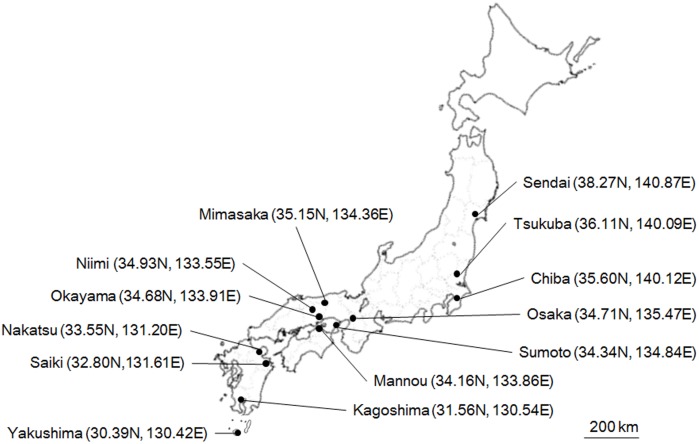
Collection points (filled circles) of *Z*. *maha* in Japan. The numerals in parentheses after the population name show the latitude and longitude, respectively.

We collected eight to 31 *Z*. *maha* males at thirteen points with different latitudes and longitudes in the Japanese temperate region in 2014: Sendai (38.27N, 140.87E, Aug. n = 21), Tsukuba (36.11N, 140.09E, June n = 30; Aug. n = 29; Oct. n = 24), Chiba (35.60N, 140.12E, Oct. n = 17), Osaka (34.71N, 135.47E, Oct. n = 25), Sumoto (34.34N, 134.84E, Oct. n = 27), Mimasaka (35.15N, 134.36E, July n = 25; Oct. n = 27), Niimi (34.93N, 133.55E, Oct. n = 20), Okayama (34.68N, 133.91E, June n = 26, Aug. n = 28, Oct. n = 26), Mannou (34.16N, 133.86E, Aug. n = 11, Oct. n = 25), Nakatsu (33.55N, 131.20E, Aug. n = 25, Oct. n = 24), Saiki (32.80N, 131.61E, Sept. n = 12, Oct. n = 8), Kagoshima (31.56N, 130.54E, Apr. n = 15, Oct. n = 17), and Yakushima (30.39N, 130.42E, Oct. n = 23). The geographic distribution of each sampling point is shown in Fig 1. These collection sites covered an extensive habitat of Japan, except Hokkaido Island, the northern part of Japan, where this species is not distributed. We also did not collect from the Okinawa Islands where a sub-species is distributed.

We collected flying adults by butterfly net, and the collected samples were almost all males (more than 90%, T. Sumi, unpublished data) because almost all flying adults were male. Thoraces width of collected samples was measured their thorax width under a stereomicroscope. After that, the samples were stored in 99.5% ethanol. We investigated the *Wolbachia* strains that infected *Z*. *maha*.

### DNA extraction

DNA was extracted from each specimen’s abdomen (excluding the first to the third sternites). The whole abdomen including gonads and testes was crushed by a toothpick in an Eppendorf tube (1.5ul) with Chelex^®^ 100 (Bio-Rad) 98ul+Proteinase K (TaKaRa). The samples were incubated three hours at 56°C (spinning samples from 6 to 8 times each 30 min) to dissolve protein, and then they were incubated in a heat block set at 100°C for 3 min. After that, the sample was cooled to 5°C, and stored at -20°C.

We used CO1 primers [15] to confirm DNA by PCR. After confirmation of DNA, we used *wsp* primers to investigate *Wolbachia* infection [16]. To investigate their sequence, we used *Wolbachia* strains that infected *Z*. *maha* as host, *Wolbachia* MLST primers [13], CO1 [16], and *wingless* primers [17]. CO1 and *wingless* primers were used to investigate the phylogenetic relationships of samples. *Wolbachia* causes a selective sweep that shows confused genetic link between nuclear DNA and mitochondrial DNA. Accordingly, we selected *wingless* as a nuclear gene marker. Sequence analyses were carried out by Fasmac Co. Ltd. (Kanagawa, Japan). *Wsp*, CO1, and *wingless* gene sequences were assembled using DNA Baser version 4.2 (Heracles BioSoft, Pitesti, Romania), and aligned by Mega 5 (Accession numbers: LC125171–LC125181).

We developed qPCR primers from analyzed DNA sequences. We amplified the sample of Sendai No. 1, which was the standard of qPCR in this study, by PCR of each *wsp* or CO1 primer pair. After that, PCR products were diluted 10 times in 10 steps. For real-time qPCR, we used iQ SYBR Green Supermix (Biorad, Tokyo) and followed the supplier’s protocol. Then, we obtained standard curves of CO1 and *wsp* from the standard sample. Next, we amplified each sample DNA by qPCR on CO1 and *wsp* primer pairs, and qPCR cycles were fitted by each standard curve. Then we obtained copies of each CO1 and *wsp* gene. We considered that the butterfly cells were approximated by CO1 gene copies. Therefore, to evaluate *Wolbachia* density, each gene copy number was calculated by a standard curve, and *wsp* was normalized to CO1. All primers are shown in electronic supplementary S2 Table. We always used Sendai No. 1 as the standard for creating this standard curve.

To compare *Wolbachia* infection rates amongst each geographical population, Fisher’s exact test was used. *Wolbachia* densities in each population were tested by a Kruskal-Wallis rank sum test, and followed by a Steel-Dwass test (referenced by http://aoki2.si.gunma-u.ac.jp/R/src/Steel-Dwass.R). To determine the relationship between *Wolbachia* density and collection season, we used a linear model from the eight points that were collected over several months. In each model, collection month and *Wolbachia* density were used as explanatory and response variables, respectively, for the eight points. Then, the models were tested by ANOVA. To investigate the correlation between latitude or longitude and *Wolbachia* density, we used the Pearson product-moment correlation coefficient. All statistical analyses were performed using R version 3.1.2 [18]

Our butterfly collection sites did not require specific permissions to catch the invertebrates. Moreover, this butterfly is not protected by any law. Further, we do not conflict with any guideline of PLoS ONE because we used only the butterfly *Z*. *maha*.

### Reproductive manipulation

To determine the type of *Wolbchia* infecting the butterfly, i.e., to examine the control of reproduction by *Wolbachia* infection, seven females collected from Okayama City in 2015 were allowed oviposit on the leaves of *Oxalis corniculata*. The larvae hatched from the eggs were divided into two groups, i.e., larvae were provided leaves grown in DW with 0.5% tetracycline (Tc) or without tetracycline (C), namely in DW only. Larvae were reared in the laboratory at 25°C, RH 60%, and 16L:8D dark-light cycles, and a laboratory population was established. Adults emerging via pupae from these larvae were reared in a hydroponic culture system (Green Farm UH-A01E1: U-ING, Osaka, Japan) in which *Oxalis corniculata* were planted. The larvae of the 1st and 2nd generations were reared on food including tetracycline (Tc) or not including tetracycline (C). Then, the larvae of 3rd and 4th generations were reared on *Oxalis corniculata* alone to eliminate the effect of tetracycline.

At the fourth successive generation, reciprocal crossing was conducted; i.e., females of Tc groups were paired with Tc and C males, and females of C groups were paired with Tc and C males. Each pair was allowed to copulate for two weeks for all crosses. The numbers of eggs oviposited by these females (n = 3 for each cross) and hatching rates were counted.

## Results

### Field population

**Fig 2:**
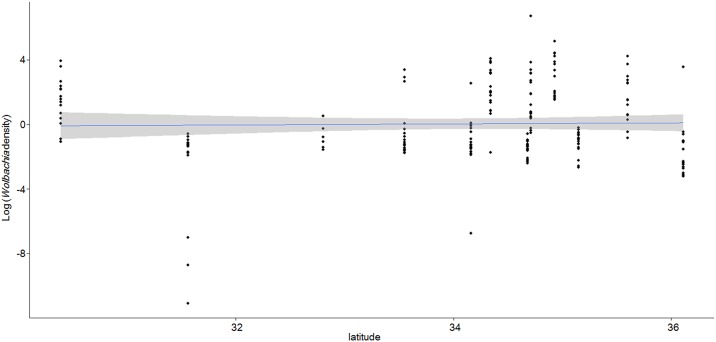
Relationship between latitude and *Wolbachia* density. *Wolbachia* density of all individuals is plotted by latitude. Solid lines show a liner model of *Wolbachia* density by latitude. The grey zone shows the 95% confidence interval.

MLST revealed that the *Wolbachia* strain did not differ amongst the *Z*. *maha* populations (LC125171–LC125176). The *Wolbachia* alleles revealed by the MLST analysis included coxA 14, fbpB 4, ftsZ 36, gatB 39, and hcpA 40. Therefore, the *Wolbachia* type was identified as ST41 by *Wolbachia* MLST Databases (https://pubmlst.org/wolbachia/). In the host genotype, the CO1 sequence differed only in Sendai (LC1251810). Similarly, the *wingless* sequence differed from other populations in Kagoshima (LC125179) and Tsukuba (LC12577). Numbers of infected adults with the numbers collected and tested for *Wolbachia* density, indexes of density of *Wolbachia* infection, and body sizes of *Z*. *maha* are shown in the S1 Table. *Wolbachia* infection rates were not significantly different amongst populations in all monthes examined (*P* > 0.05). However, *Wolbachia* infection density significantly differed amongst populations collected in October (S1 Fig, Tsukuba, 0.1, 0.04, 34.62; Chiba, 4.46, 0.43, 67.34; Osaka, 2.78, 0.585, 811.86; Sumoto, 7.45, 0.18, 58.93; Mimasaka, 0.38, 0.07, 0.82; Niimi, 13.73, 4.62, 171.65; Okayama, 0.25, 0.09, 0.55; Mannou, 0.27, 0.001, 12.75; Nakatsu, 0.36, 0.17, 29.55; Saiki, 2.08, 0.19, 52.14; Kagoshima, 0.27, 0.00002, 0.55; Yakushima, 5.67, 0.34, 51.65, median, minimum and maximum density each), although no correlation was found between the latitude or longitude of collection sites and *Wolbachia* density in October (latitude: Fig 2, r = 0.04, *P* = 0.569, longitude: S2 Fig, r = 0.062, *P* = 0.376).

**Fig 3:**
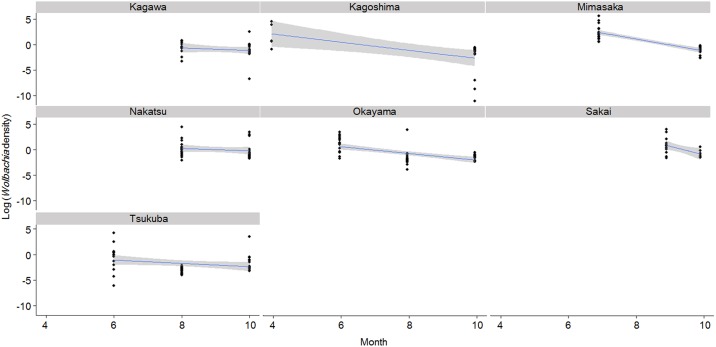
Relationship between month collected and *Wolbachia* density at each collection site. A solid line shows a linear model of *Wolbachia* density by month at each site. The grey zone shows the 95% confidence interval.

To investigate whether the change of season affected *Wolbachia* density, we compared *Wolbachia* densities by collection month using a Steel-Dwass test. As a result, *Wolbachia* density significantly differed between two seasons in each population in 2014 (Tsukuba: June vs. Aug., *P* = 0.001; June vs. Oct., *P* = 0.07; Aug. vs. Oct., *P* = 0.001, Mimasaka, *P* < 0.001, Okayama: June vs. Aug., *P* < 0.001; June vs. Oct., *P* < 0.001; Aug. vs. Oct., *P* = 0.073, Mannou, *P* = 0.16, Nakatsu, *P* = 0.055, Saiki, *P* = 0.028). Then we examined whether the same seasonal change in *Wolbachia* density occurred in each population. Statistical analysis showed that *Wolbachia* density increased in early summer and decreased in autumn. However, there was a significant difference in the effects of month and population for each population, and the month × population interactions was also significantly different (Fig 3, month: *P* < 0.01, population: *P* < 0.01, month × population: *P* < 0.005).

### Reproductive manipulation

The numbers of eggs oviposited were 906 for C females×C males, 849 for C females × Tc males, 689 for Tc females × C males, and 809 for Tc females × Tc males. Eggs successfully hatched were 810 (89.4%), 733 (86.3%), 3 (0.4%), and 705 (87.1%) for C × C, C×Tc, Tc × C, and Tc×Tc, respectively (*P* < 0.001, Fisher’s exact test). The results indicate that the *Wolbachia* strain infecting *Z*. *maha* causes cytoplasmic incompatibility.

## Discussion

MLST conducted in the present study revealed that the *Wolbachia* strain infecting *Z*. *maha* was ST41. ST41 is prevalent in many butterfly species [19, 20] and causes cytoplasmic incompatibility (CI), as shown in the present results by reproductive manipulation [21]. No seasonal difference was observed in the infection rates of *Wolbachia*. However, we found that *Wolbachia* densities fluctuate seasonally. The density was significantly higher from spring (Apr to June) to early summer (July) than in late summer (Aug), and it decreased during late summer and autumn (Sept and Oct) (Fig 3, S1 Table). Compared with median *Wolbachia* density in population by season, the highest difference was 89 times between from spring (24.08) to autumn (0.27) in Kagoshima, and from late summer to autumn the difference was around two times in Tsukuba and Okayama. Accordingly, the seasonal fluctuation of *Wolbachia* density in hosts might be affected by the environment around the host habitat. It has been reported that *Wolbachia* density varied significantly between seasons within a population by annual observation of *D*. *innubila* [10]. Unckless et al. (2009) suggested that individuals with a lower *Wolbachia* density were older than individuals that had a high density [10]. However, the adult longevity of *Z*. *maha* is short (about one to two weeks in laboratory conditions), and the change of generations is about one month [22]. Therefore, we do not think that only older individuals were collected in the late summer (the season of lowest *Wolbachia* density).

Although *Wolbachia* densities in *Z*. *maha* were significantly different amongst populations, a geographical cline of *Wolbachia* density was not observed. In the present result, the “geographical cline of *Wolbachia* density hypothesis” was dismissed. By median *Wolbachia* density, the highest population (Niimi) was 137 times higher than the lowest population (Tsukuba) in October. Surprisingly, *Wolbachia* density has an extremely wide range in natural population and at least difference of six times in density in the present study. On the other hand, *Wolbachia* density had small range in previous studies in the laboratory (e.g. Mouton et al., 2007). This result suggests that environmental factors are related to the seasonal changes of *Wolbachia* density in the Japanese temperate region. Hence, we suggest that temperature is the most significant factor in seasonal changes of *Wolbachia* density in populations in a temperate region.

Given that *Wolbachia* is a maternally-transmitted bacterium, seasonally-induced changes in *Wolbachia* density are likely more important in female insects than in males. However, we collected mostly males in this study, as described in the introduction. Therefore, additional studies using female samples are required. Because it is difficult to collect flying females in the field, analyzing samples of the larvae or pupae collected from fields is required in the future. However, we consider that it is suitable to use males in our study. Because of this, we revealed this *Wolbachia* strain was characterized as ST41, which causes CI. Furthermore, CI was caused by males.

An interaction between food quality and *Wolbachia* density was revealed by several studies in the laboratory. For example, *Wolbachia* density in *Aedes albopictus* was changed by the host juvenile diet [23], although the change in *Wolbachia* density was reported only in females [23]. Moreover, another study reported that food quality did not affect *Wolbachia* density in *Drosophila innubila* [10]. When *Wolbachia* density is high, it strongly manipulates host reproduction. Why a good nutrient condition did not affect *Wolbachia* density is unclear. If *Wolbachia* density in *Z*. *maha* males is not also changed by nutrient conditions, it suggests efficient *Wolbachia* transmission from mother and environment included seasonal nutrients affecting *Wolbachia* density (see S3 Fig). On the other hand, not only environmental conditions but genetic mechanisms of the host regulate *Wolbachia* density [11]. Furthermore, other endosymbionts may affect regulation of *Wolbachia* density [23]. In particular, it is possible that the difference in *Wolbachia* density amongst populations corresponds to competition between *Wolbachia* and other endosymbionts. In a future study, we hope to determine whether *Z*. *maha* is infected with other endosymbionts that compete with *Wolbachia*.

The present study confirmed that the rate of *Wolbachia* infection in *Z*. *maha* did not differ amongst populations in Japan, as reported in our previous study [13]. This result also suggests that *Wolbachia* strongly affect host reproduction of *Z*. *maha*. Furthermore, if *Wolbachia* infection strongly affects other aspects of host performance, it is shown in the following examples in *Z*. *maha*. For example, Charlat et al. (2007) reported that sudden male killing by *Wolbachia* did not manipulate *Hypolimnas bolina*. Similarly, *Armadillidium vulgare* have a resistance factor to feminization by *Wolbachia* [24]. These results suggest that if *Z*. *maha* does not suffer costs from *Wolbachia* infection, infection rates may change in the future. We considered further experimentation is required to unravel this issue. On the other hand, *Wolbachia* protect their host from virulent RNA viruses [25–27]. One reason for the high infection rate by *Wolbachia* of *Z*. *maha* is that it provides some benefit to the host.

In conclusion, our results provide the first evidence that *Wolbachia* density in *Z*. *maha* populations differs between seasons in the field and is higher in spring than in summer. However, the *Wolbachia* infection rate of this species did not change. This seasonal change may be prevalent in wild invertebrates infected by *Wolbachia*.

## Supporting information

S1 Fig Comparison of *Wolbachia* densities in each *Z*. *maha* population in October. An open circle above a box plot indicates an outlier. A different letter shows a significant difference (*P* < 0.05, Steel-Dwass test).
(TIF)

S2 Fig Relationship between longitude and *Wolbachia* density. *Wolbachia* density of all individuals is plotted by longitude. A solid line shows a linear model of *Wolbachia* density by longitude. The grey zone shows the 95% confidence interval.
(TIF)

S3 Fig Relationships between thorax width and *Wolbachia* density. *Wolbachia* density of each population was plotted by host thorax width. The solid line shows a linear model of *Wolbachia* density by host thorax width. The grey zone shows the 95% confidence interval.
(TIF)

S1 Table The numbers collected, infected and tested for Wolbachia density, indexes of density of *Wolbachia* infection, and body sizes of *Z*. *maha* collected from fields. (XLSX)

S2 Table The primers used in this experiment. (XLSX)

## References

[1] O’Neill SL, Hoffman AA, Werren JH. 1997 Influential Passengers: Inherited Microorganisms and Arthropod Reproduction. Oxford university Press

[2] Werren JH, Baldo L, Clark ME. 2008 *Wolbachia*: master manipulators of invertebrate biology. Nat Rev Microbiol 6: 741–751 10.1038/nrmicro1969 18794912

[3] Stouthamer R, Luck RF, Hamilton WD. 1990 Antibiotics cause parthenogenetic Trichogramma (Hymenoptera, Trichogrammatidae) to revert to sex. PNAS 87:2424–2427 11607070 10.1073/pnas.87.7.2424 PMC53701

[4] Werren JH. 1997 Biology of *Wolbachia*. Annu. Rev. Entomol. 42: 587–609 15012323 10.1146/annurev.ento.42.1.587

[5] Perrot-Minnot MJ, Guo LR, Werren JH. 1996 Single and double infections with *Wolbachia* in the parasitic wasp *Nasonia vitripennis*: effects on compatibility. Genetics 143:961–972 8725242 10.1093/genetics/143.2.961 PMC1207352

[6] Johanowicz DL, Hoy MA. 1998 Experimental induction and termination of non-reciprocal reproductive incompatibilities in a parahaploid mite. Entomol Exp Appl 87:51–58

[7] Van Opijnen T, Breeuwer JAJ. 1999 High Temperatures Eliminate *Wolbachia*, a cytoplasmic incompatibility inducing endosymbiont, from the two-spotted spider mite. Exp Appl Acarol 23: 871–881 10668862 10.1023/a:1006363604916

[8] Mouton L, Henri H, Bouletreau M, Vavre F. 2006 Effect of temperature on *Wolbachia* density and impact on cytoplasmic incompatibility. Parasitology 132:49–56 16393353 10.1017/S0031182005008723

[9] Duron O, Labbé P, Berticat C, Rousset F, Guillot S, Raymond M, Weill M. 2006 High *Wolbachia* density correlates with cost of infection for insecticide resistant *Culex pipiens* mosquitoes. Evolution 60(2): 303–314 16610322

[10] Unckless RL, Boelio LM, Herren JK, Jaenike J. 2009 *Wolbachia* as populations within individual insects: causes and consequences of density variation in natural populations. Proc. R. Soc. B 276: 2805–2811 10.1098/rspb.2009.0287 19419989 PMC2839946

[11] Mouton L, Henri H, Charif D, Boulétreau M, Vavre F. 2007 Interaction between host genotype and environmental conditions affects bacterial density in *Wolbachia* symbiosis. Biol Lett 3: 210–213 10.1098/rsbl.2006.0590 17251124 PMC2375926

[12] Toju H, Fukatsu T. 2011 Diversity and infection prevalence of endosymbionts in natural populations of the chestnut weevil: relevance of local climate and host plants. Mol. Ecol. 20, 853–868 10.1111/j.1365-294X.2010.04980.x 21199036

[13] Sumi T, Miura K, Miyatake T. 2013 No seasonal trend in infection of the pale grass blue butterfly, *Zizeeria maha* (Lepidoptera: Lycaenidae), by *Wolbachia*. Appl Entomol Zool 48: 35–38

[14] Bald L, Hotopp JCD, Jolley KA, Bordenstein SR, Bilber SA, Choudhury RR et al 2006 Multilocus Sequence Typing System for the Endosymbiont *Wolbachia pipientis*. Appl. Environ. Microbiol. 72(11): 7098–7110 10.1128/AEM.00731-06 16936055 PMC1636189

[15] Folmer O, Black M, Hoeh W, Lutz R, Vrijenhoek R. 1994 DNA primers for amplification of mitochondrial cytochrome c oxidase subunit I from diverse metazoan invertebrates. Mol Mar BiolBiotech 3: 294–299 7881515

[16] Braig HR, Zhou W, Dobson SL, O’Neill SL. 1998 Cloning and characterization of a gene encoding the major surface protein of the bacterial endosymbiont *Wolbachia pipientis*. J Bacteriol 180: 2373–2378 9573188 10.1128/jb.180.9.2373-2378.1998 PMC107178

[17] Wild AL, Maddison DR. 2008 Evaluating nuclear protein-coding genes for phylogenetic utility in beetles. Mol. phylogenet. Evol. 48(3): 877–891. 10.1016/j.ympev.2008.05.023 18644735

[18] R Core Team (2014). R: A language and environment for statistical computing. R Foundation for Statistical Computing, Vienna, Austria.

[19] Hiroki M, Tagami Y, Miura K, Kato Y. 2004 Multiple infection with Wolbachia inducing different reproductive manipulations in the butterfly *Eurema hecabe*. Proc R Soc B: 271, 1751–1755 10.1098/rspb.2004.2769 15306297 PMC1691781

[20] Salunke BK, Salunkhe RC, Dhotre DP, Walujkar SA, Khandagale AB, Chaudhari R et al 2012 Determination of *Wolbachia* diversity in butterflies from Western Ghats, India, by a multigene approach. Appl Environ Microbiol 78: 4458–4467 10.1128/AEM.07298-11 22504801 PMC3370507

[21] Narita S, Kageyama D, Nomura M, Fukatsu T. 2007 Unexpected mechanism of symbiont-induced reversal of insect sex: feminizing Wolbachia continuously acts on the butterfly Eurema hecabe during larval development. Appl Environ Microbiol 73, 4332–4341 10.1128/AEM.00145-07 17496135 PMC1932763

[22] Hiyama A, Nohara C, Taira W, Kinjo S, Iwata M, Otaki JM. 2013 The Fukushima nuclear accident and the pale grass blue butterfly: evaluating biological effects of long-term low-dose exposures. BMC Evol Biol 13: 168 10.1186/1471-2148-13-168 23937355 PMC3751199

[23] Dutton TJ and Sinkins SP. 2004 Strain-specific quantification of *Wolbachia* density in *Aedes albopictus* and effects of larval rearing conditions. Insect Mol. Biol. 13: 317–322 15157232 10.1111/j.0962-1075.2004.00490.x

[24] Charlat S, Hornett EA, Fullard JH, Davies N, Roderick GK, Wedell N et al 2007 Extraordinary Flux in Sex Ratio. Science 317: 214 17626876 10.1126/science.1143369

[25] Rigaud T, Juchault P. 1992 Genetic control of the vertical transmission of a cytoplasmic sex factor in *Armadillidium vulgare* Latr. (Crustacea, Oniscidea). Heredity 68: 47–52

[26] Hedges LM, Brownlie JC, O’Neill SL, Johnson KN. 2008 *Wolbachia* and Virus Protection in Insects. Science 322: 702 10.1126/science.1162418 18974344

[27] Teixeira L, Ferreira Á, Ashburner M. 2008 The Bacterial Symbiont *Wolbachia* Induces Resistance to RNA Viral Infections in *Drosophila melanogaster*. PLoS Biol. 6: 2753–2763 10.1371/journal.pbio.1000002 PMC2605931 19222304

