# Supplementary figures and images for: Wolbachia density changes seasonally amongst populations of the pale grass blue butterfly, Zizeeria maha (Lepidoptera: Lycaenidae)
Source: PLoS One. 2017 Apr 12;12(4):e0175373. doi: 10.1371/journal.pone.0175373 (PMC5389786; doi:10.1371/journal.pone.0175373)

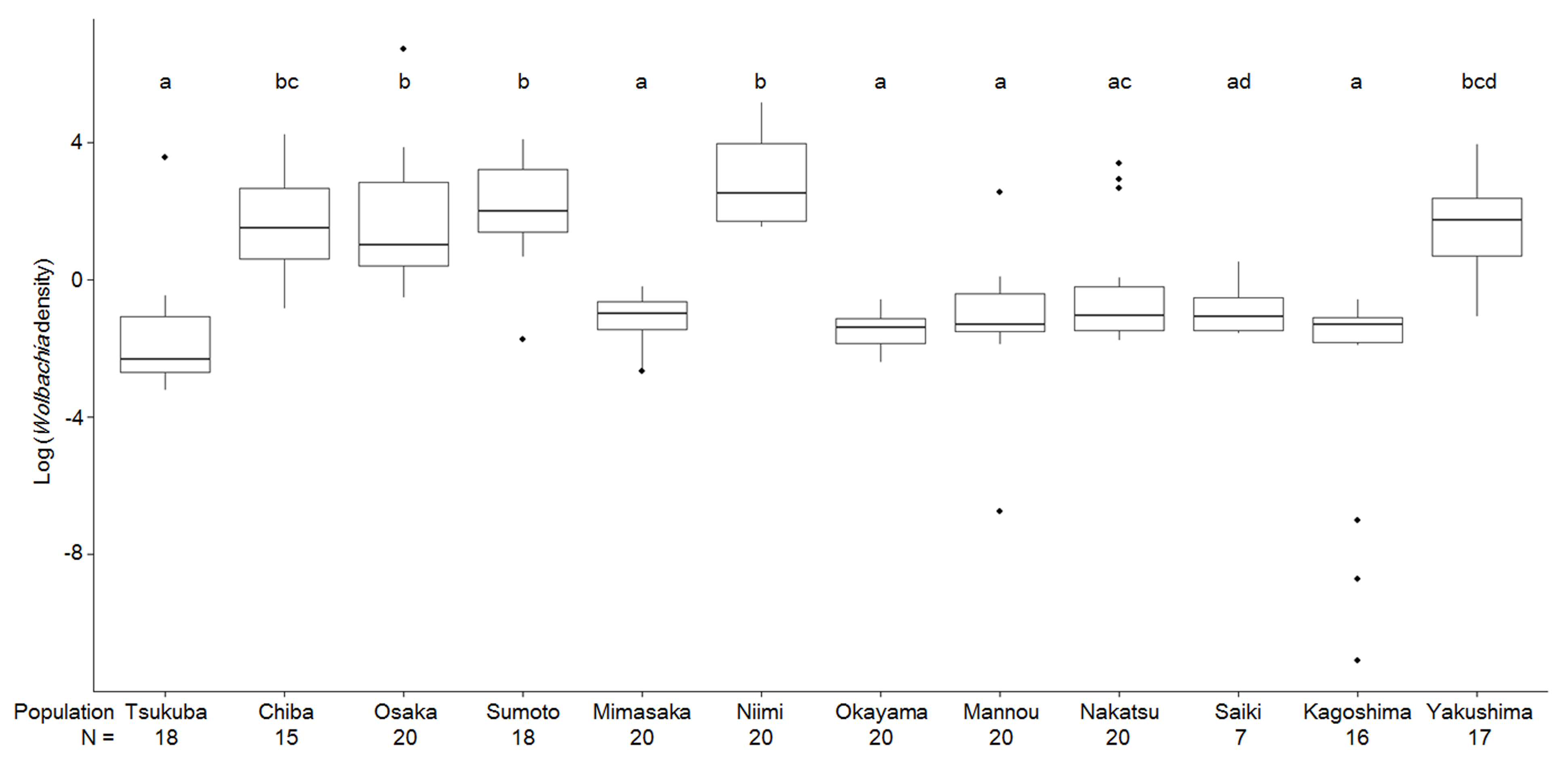

Supplement: S1 Fig — An open circle above a box plot indicates an outlier. A different letter shows a significant difference (P < 0.05, Steel-Dwass test). (TIF) [file pone.0175373.s001.tif]

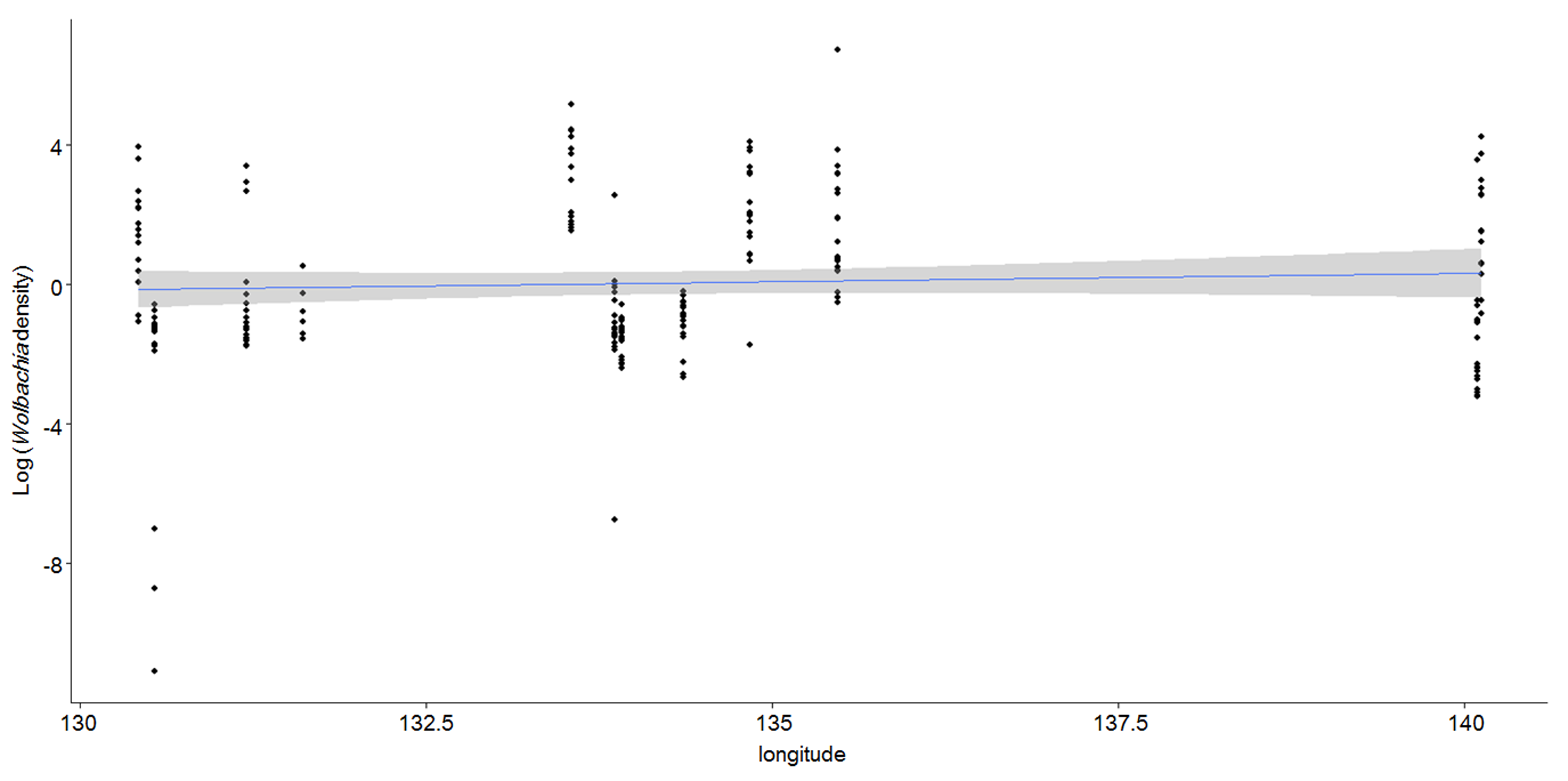

Supplement: S2 Fig — Wolbachia density of all individuals is plotted by longitude. A solid line shows a linear model of Wolbachia density by longitude. The grey zone shows the 95% confidence interval. (TIF) [file pone.0175373.s002.tif]

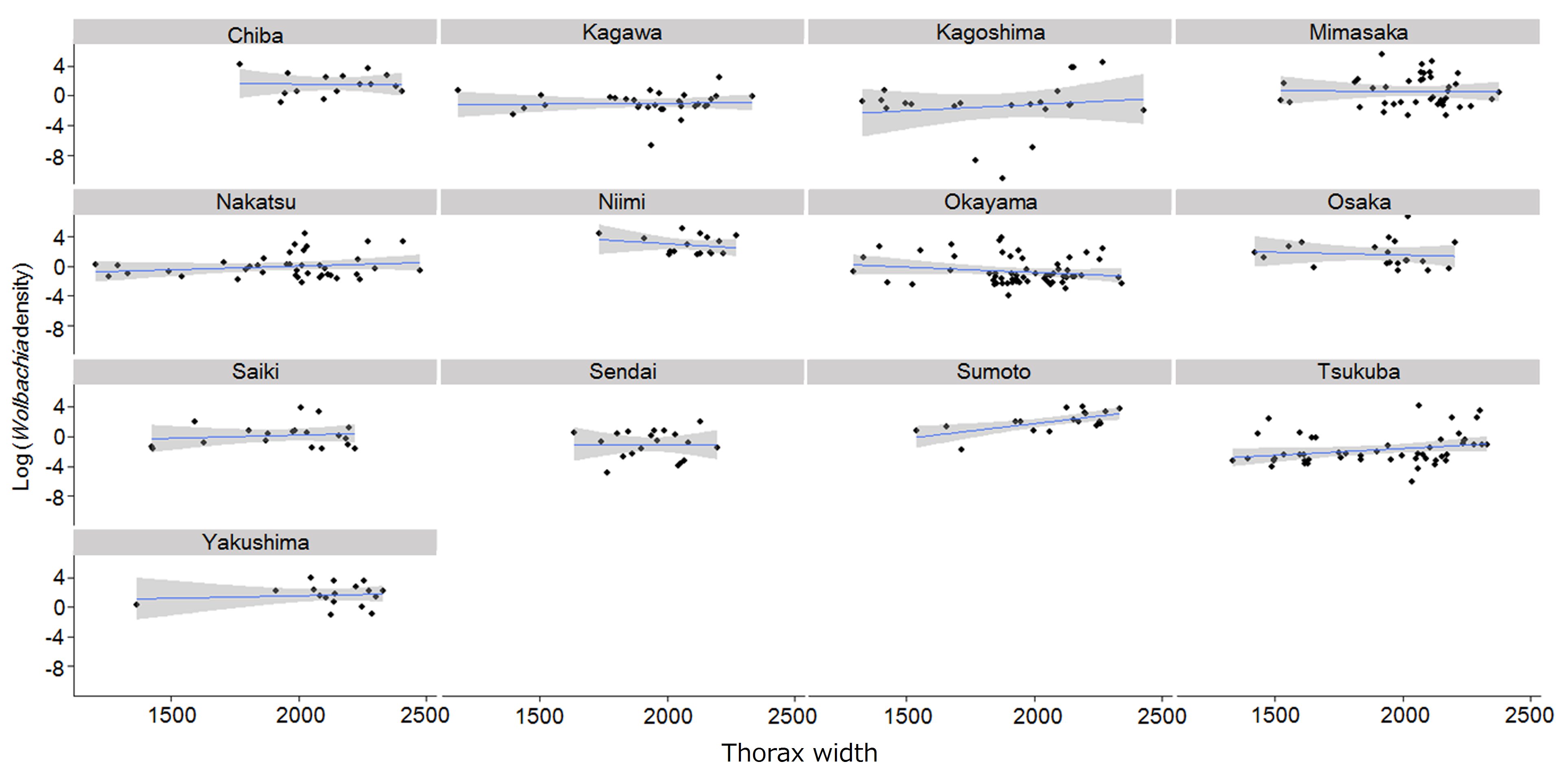

Supplement: S3 Fig — Wolbachia density of each population was plotted by host thorax width. The solid line shows a linear model of Wolbachia density by host thorax width. The grey zone shows the 95% confidence interval. (TIF) [file pone.0175373.s003.tif]
